# Photosynthetic Conversion of Carbon Dioxide to Oleochemicals by Cyanobacteria: Recent Advances and Future Perspectives

**DOI:** 10.3389/fmicb.2020.00634

**Published:** 2020-04-17

**Authors:** Li Wang, Liyuan Chen, Shihui Yang, Xiaoming Tan

**Affiliations:** State Key Laboratory of Biocatalysis and Enzyme Engineering, Environmental Microbial Technology Center of Hubei Province, School of Life Sciences, Hubei University, Wuhan, China

**Keywords:** cyanobacterium, oleochemicals, metabolic engineering, carbon dioxide conversion, lipid metabolism

## Abstract

Sustainable production of biofuels and biochemicals has been broadly accepted as a solution to lower carbon dioxide emissions. Besides being used as lubricants or detergents, oleochemicals are also attractive biofuels as they are compatible with existing transport infrastructures. Cyanobacteria are autotrophic prokaryotes possessing photosynthetic abilities with mature genetic manipulation systems. Through the introduction of exogenous or the modification of intrinsic metabolic pathways, cyanobacteria have been engineered to produce various bio-chemicals and biofuels over the past decade. In this review, we specifically summarize recent progress on photosynthetic production of fatty acids, fatty alcohols, fatty alk(a/e)nes, and fatty acid esters by genetically engineered cyanobacteria. We also summarize recent reports on fatty acid and lipid metabolisms of cyanobacteria and provide perspectives for economic cyanobacterial oleochemical production in the future.

## Introduction

Since the Industrial Revolution, the level of global carbon dioxide together with other greenhouse gases (GHGs) significantly increased due to human activities (Ainsworth et al., 2020). Cumulative anthropogenic emissions of CO_2_ have been considered as the main driver of global warming (Ainsworth et al., 2020). Transportation is a major contributor to the global CO_2_ emission, representing 65% of the world oil consumption and 24% of global CO_2_ emissions due to the direct combustion of fuels (Li et al., 2019; Solaymani, 2019). With the worldwide concerns about global warming, biofuels have been embraced as promising alternatives to fossil fuels, because they are renewable and generally can lower carbon emissions (Demirbas, 2009; Gaurav et al., 2017).

Oleochemicals are a large group of fatty acid derivatives, including fatty acids, fatty alcohols, fatty alk (a/e)nes, and fatty acid methyl/ethyl esters and waxes (Pfleger et al., 2015). They can be used as biodiesels, lubricants, and surfactants, and others (Yu et al., 2014; Pfleger et al., 2015; Marella et al., 2018). Compared with ethanol, which is another popular biofuel molecule, lipid-derived biodiesels have been considered to be better biofuel molecules due to their high energy density and compatibility with the existing liquid fuel infrastructure (i.e., fuel engines, refinery equipment, and transportation pipelines) (Lu, 2010).

**FIGURE 1:**
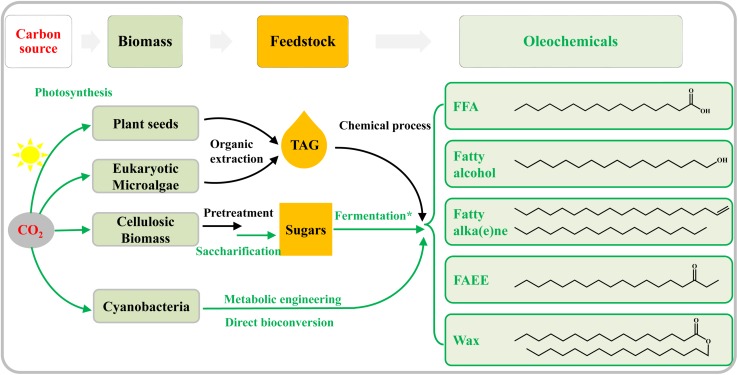
Overview of both traditional and emerging technologies for the bio-production of oleochemicals. The green lines show enzymatic or biological conversion, whereas physical or chemical processes are in black. Vegetable oil seeds are traditionally utilized to produce oleochemicals. Oleaginous eukaryotic microalgae are attractive alternatives to plant oil. Abundant cellulosic biomass is first hydrolyzed to sugars, and the latter is then fermented to produce oleochemicals by the engineered heterotrophic microbes (indicated by the asterisk). Harboring photosynthesis ability, cyanobacteria can be genetically modified to direct convert CO_2_ to oleochemicals.

Traditionally, crop oils and animal fats (Figure 1) were used as feedstocks for the production of oleochemicals by chemical or enzymatic processes (Pfleger et al., 2015). However, this traditional route for oleochemical production will compete with crops for arable land, decrease food production, and raise serious concerns about food security (Graham-Rowe, 2011). Microalgae have been considered as promising feedstocks for oleochemicals (Figure 1) because of their higher lipid productivities per ground area than oleaginous agricultural crops, as well as the lack of competition they would provide for agricultural land (Mata et al., 2010; Wijffels and Barbosa, 2010). Besides, abundant lignocellulosic biomass has become another ideal feedstock for the production of oleochemicals (Figure 1), in the context of large-scale metabolic engineering efforts in microbial systems (Lee et al., 2008; Alper and Stephanopoulos, 2009; Peralta-Yahya and Keasling, 2010; Keasling, 2012). Some heterotrophic model microorganisms, such as *Escherichia coli* and *Saccharomyces cerevisiae*, have been genetically modified to produce many kinds of biofuels and bio-chemicals including oleochemicals from lignocellulosic sugars (Atsumi et al., 2008; Steen et al., 2010; Buijs et al., 2013). Some recent review articles have summarized biosynthesis pathways, metabolic engineering strategies, and challenges for the production of oleochemicals by heterotrophic microbes (Janssen and Steinbuchel, 2014; Pfleger et al., 2015; Marella et al., 2018).

Cyanobacteria are the only prokaryotes capable of performing oxygen-evolving photosynthesis (Hagemann and Hess, 2018), and have been the genetic models for photosynthesis research for decades (Wang et al., 2018). They were initially not considered to be useful to the Aquatic Species Program for biofuel production, because most of them do not naturally accumulate storage lipids in the form of triacylglycerol (TAG) as some oleaginous eukaryotic microalgae do (Sheehan et al., 1998). However, cyanobacteria have emerged as novel chassis strains for the production of biofuels and bio-chemicals since 2009, owing to their photosynthetic abilities and reliable genetic systems (Angermayr et al., 2009; Atsumi et al., 2009; Dexter and Fu, 2009; Lindberg et al., 2009). Engineered cyanobacteria are able to produce various compounds directly from CO_2_, bypassing the need for fermentable sugars and arable land (Lai and Lan, 2015). In the past decade, photosynthetic production of various compounds, including oleochemicals (Figure 1), has been achieved in several model cyanobacteria through metabolic engineering (Zhou and Li, 2010; Angermayr et al., 2015; Savakis and Hellingwerf, 2015; Oliver et al., 2016; Xiong et al., 2017). This review summarizes current knowledge on the metabolism of fatty acids and membrane lipids in cyanobacteria, provides the current status of metabolic engineering strategies for producing oleochemicals, and discusses key challenges and possible solutions in the field.

## Metabolisms of Fatty Acids and Membrane Lipids in Cyanobacteria

The biosynthesis of membrane lipids in cyanobacteria has been investigated since the 1980s (Naoki and Norio, 1982) and was followed by systematical works by Murata and co-workers in the 1990s (Wada and Murata, 1990; Wada and Murata, 1998) and 2000s (Sato and Wada, 2009). Unlike heterotrophic prokaryotes, the vast majority of cyanobacteria have thylakoid membranes in their cytoplasm where photosynthesis takes place (Rexroth et al., 2011). Both cytoplasmic (plasma) and thylakoid membranes of cyanobacteria include four major polar glycerolipids: monogalactosyl diacylglycerol (MGDG), digalactosyl diacylglycerol (DGDG), sulfoquinovosyldiacylglycerol (SQDG), and phosphatidylglycerol (PG) (Los and Mironov, 2015). Despite a report indicating the occurrence of neutral lipid droplets including triacylglycerol (TAG) in the cyanobacterium *Nostoc punctiforme* PCC73102 (hereafter Npu73102) (Peramuna and Summers, 2014), it is noteworthy that the above four polar lipids still serve as the dominant sink for fatty acids in cyanobacteria.

### Fatty Acid Biosynthesis Pathway

**FIGURE 2:**
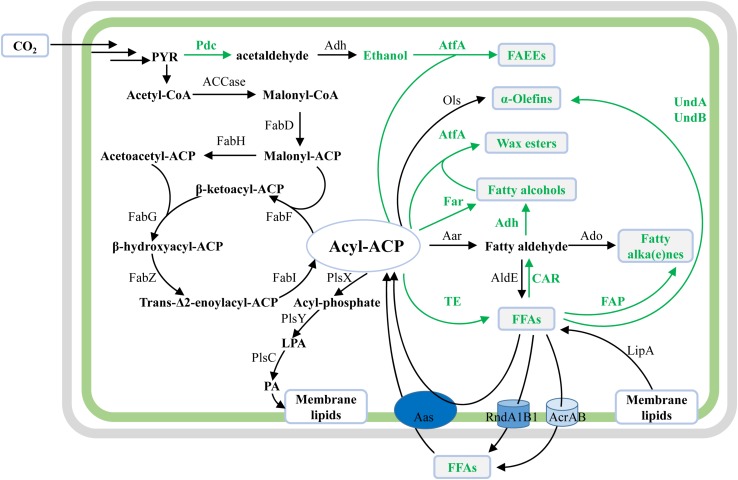
Schematic overview of native and synthetic pathways for biosynthesis of membrane lipids and oleochemicals in cyanobacteria. The illustration shows the native biosynthesis pathways for membrane lipids, together with synthetic pathways for cyanobacterial oleochemical production. For synthetic pathways, enzymes and reaction direction are shown in green. For native cyanobacterial pathways, enzymes and reaction direction are in black. Oleochemicals discussed in this work are shown in green texts and in gray boxes.

Same as the widely studied fatty acid biosynthesis pathway in *E. coli*, cyanobacterial fatty acid biosynthesis pathways are composed of reactions catalyzed by two protein complexes, namely, acetyl-CoA carboxylase (ACCase) and type II fatty acid synthase (FAS) encoded by *fab* genes (Sato and Wada, 2009). In brief, acetyl-CoA is firstly converted to malonyl-CoA by acetyl-CoA carboxylase, and then to malonyl-ACP by malonyl-CoA:ACP transacylase (FabD) (Figure 2). Subsequently, butyryl-ACP is generated by sequential reactions catalyzed by β-ketoacyl-ACP synthase III (FabH), β-ketoacyl-ACP reductase (FabG), β-hydroxyacyl-ACP dehydrase (FabZ), and enoyl-ACP reductase (FabI). The fatty acid chain is then elongated with an acetyl unit from malonyl-ACP for each condensation-reduction-dehydration-reduction cycle (Figure 2) (Sato and Wada, 2009). For most cyanobacteria, palmitoyl-ACP (C16) and stearoyl-ACP (C18) are used as precursors for the biosynthesis of membrane lipids. Contrary to previous findings in *E. coli* (Yu et al., 2011), it was proved that FabH, which condenses malonyl-ACP with acetyl-CoA to form acetoacetyl-ACP, is the sole rate-limiting enzyme of FAS in *Synechococcus* sp. PCC7002 (hereafter Syn7002) (Kuo and Khosla, 2014).

### Metabolisms of Membrane Lipids

For the biosynthesis of the four polar glycerolipids mentioned above, phosphatidic acid (PA) is synthesized as the common precursor by the acylation of both sn-1 and 2 positions of glycerol-3-phosphate (G3P) with the long-chain fatty acyl-A (C16 or C18) by different acyltransferases (Sato and Wada, 2009). Specifically, the fatty acyl-ACPs are first activated by an inorganic phosphate group by phosphate acyltransferase (PlsX), and subsequently transferred to the sn-1 position of G3P by acylglycerol-phosphate acyltransferase (AGPAT or PlsY), resulting in lysophosphatidic acid (LPA) (Figure 2). Secondly, lysophosphatidic acid acyltransferase (LPAAT or PlsC) catalyzes the transfer of fatty acid chains to the sn-2 position of LPA in the PA biosynthesis, resulting in PA (Figure 2). Although the over-expression of the putative PlsX enhanced lipid production in *Synechocystis* sp. PCC 6803 (hereafter Syn6803) (Towijit et al., 2018), the detailed enzymatic characteristics of both PlsX and PlsY are still unknown. Sll1848 was identified as the primary LPAAT with a high specificity for 16:0-ACP (Weier et al., 2005), whereas Sll1752 was characterized as the secondary LPAAT that prefers stearoyl and oleoyl substrates (C18) in Syn6803 (Okazaki et al., 2006) (Figure 2).

As summarized by previous reviews (Wada and Murata, 1998; Sato and Wada, 2009), different polar head groups are further transferred to the sn-3 position of PA to synthesize four major polar glycerolipids in cyanobacteria. Finally, MGDG, DGDG, SQDG, and PG have a head group of 1β-galactose, digalactose, 6-deoxy-6-sulfo-α1-glucose, and sn-glycerol 1-phosphate at their sn-3 position of the glycerol moiety, respectively, besides two acyl groups esterified at the sn-1 and sn -2 positions (Sato and Wada, 2009).

### Desaturation of Membrane Lipids

As reviewed previously, cyanobacterial desaturases were classified as acyl-lipid desaturases rather than acyl-CoA or acyl-ACP desaturases, which means the fatty acid chain would be desaturated only when fatty acids are bound to membrane lipids (Murata and Wada, 1995; Sato and Wada, 2009; Los and Mironov, 2015). In response to the cold stress, the fatty acid chains can be stepwise desaturated at the Δ9, Δ12, ω3, and Δ6 positions by four specific desaturases, namely, DesC, DesA, DesB, and DesD, respectively. The fatty acid chain length of cyanobacteria varies from C14 to C18, whereas the number of double bonds in the fatty acid chains may vary from 0 to 4, which is controlled by the activities of the above desaturases (Los and Mironov, 2015). The fatty acid composition determined by the chain length and the numbers of double bonds can be used for the classification of cyanobacterial strains (Wada and Murata, 1998).

### Regulation of Fatty Acid and Lipid Metabolisms in Cyanobacteria

As the physical barrier of cells and sites of photosynthesis and respiration, the cytoplasmic and thylakoid membranes of cyanobacteria are sensitive to various environmental stimuli. On the one hand, glycerolipid and fatty acid compositions were observed to change with alterations of growth temperature, light illumination intensity, carbon dioxide, and pH (Wada and Murata, 1990; Sakamoto and Bryant, 2002; Cuellar-Bermudez et al., 2015). On the other hand, membrane lipids play an active role in the acclimation of cyanobacteria to different environmental conditions, including high temperature (Nanjo et al., 2010) and low temperature (Murata and Wada, 1995; Sakamoto and Bryant, 2002). Despite these observations on the physiological roles of lipids, little is known about the regulation of fatty acid and lipid metabolisms in cyanobacteria.

Hik33 has been identified as the sensory histidine kinase of a two-component system which perceives the low-temperature signal and controls the expression of *desB* gene (Suzuki et al., 2000; Mikami et al., 2002; Murata and Los, 2006). Additionally, the global regulator LexA was found to repress the expression of some *fab* genes (Kizawa et al., 2017), while a transcriptional regulator, CyAbrB2, was found to inhibit the FFA production in Syn6803 (Kawahara et al., 2016). The global nitrogen regulator PII protein was shown to negatively regulate cyanobacterial fatty acid biosynthesis by transcriptional control (Verma et al., 2018) or by interacting with biotin carboxyl carrier protein (BCCP) which is a subunit of ACCase (Hauf et al., 2016).

## Engineering Cyanobacteria to Produce Free Fatty Acids

A decade ago, it was shown that the elimination of fatty acid β-oxidation by disrupting the *fadD* or *fadE* gene (Figure 2), over-expression of a thioesterase (TE) gene to release FFAs, and over-expression of ACCase have been demonstrated to be effective approaches for the overproduction of free fatty acids (FFAs) in some heterotrophic microbes like *E. coli* (Lu et al., 2008). Similar strategies were also adopted soon afterward for cyanobacterial FFA production (Table 1).

**TABLE 1:** A summary of oleochemical production by engineered cyanobacteria.

No.	Hosts	Products	Genetic modifications		Culture optimization	Titer (mg/L)	Yield^c^ (mg/g DCW)	Productivity (mg/L/h)	References
			Over-expression	Deletion					
1	Syn6803	FFA	’TesA, UcfatB1 from *U. californica*, ChfatB2 from *C. hookeriana*, CcfatB1 from *C. camphorum* and TesA137, AccBCDA	Aas, PhaAB, CphAB, Pta, S-layer protein and PBP2	1% CO_2_, 140 μE/m^2^/s	211.2	167.2^a^	ND	Liu et al., 2011a
2	Syn6803	FFA	FatB from *A. thaliana*	Aas	Air	95.1	24.5	ND	Hu et al., 2013
3	Syn6803	FFA	Membrane-located expression of ’AcTesA from *A. baylyi*		1% CO_2_, 50 μE/m^2^/s	331.0^s^	199.2^s^	1.97^s^	Afrin et al., 2018
4	Syn6803	FFA	AhFatA, AhFatB from *A. hypogaea*		Air, 40 μE/m^2^/s	ND	ND	ND	Chen et al., 2017
5	Syn6803	FFA	’TesA	Aas	1% CO_2_, 60 μE/m^2^/s	ND	209.0	ND	Yunus et al., 2018
6	Syn6803	FFA	Tes3 from *Anaerococcus tetradius*	Aas	1% CO_2_, 60 μE/m^2^/s	97.1	ND	ND	Yunus and Jones, 2018
7	Syn7942	FFA	Fat1 from *C. reinhardtii*, AccBCDA from *C. reinhardtii*, RbcLS	Aas	1% CO_2_, 60 μE/m^2^/s	23.4	155.3	0.05	Ruffing, 2013a
8	Syn7942	FFA	’TesA, RndA1B1	Aas	2% CO_2_, 180 μE/m^2^/s, overlaid with isopropyl myristate	640^s^	360.0^s^	1.48^s^	Kato et al., 2017
9	Syn7002	FFA	’TesA, RbcLS from Syn7942	Aas	1% CO_2_	131.5^s^	70.0^s^	0.27	Ruffing, 2014
10	Syn7002	FFA	UcfatB1	Aas, GlgC	1% CO_2_, 160 μE/m^2^/s	ND	ND	ND	Work et al., 2015
11	Syn7002	FFA	UcfatB1, FabH from *Chaetoceros* GSL56	Aas, FabH	1% CO_2_	ND	ND	ND	Gu et al., 2016
12	Syn6803	Alk(a/e)nes	Two copies of Ado-Aar		5% CO_2_, 100 μE/m^2^/s	26.0	11.0	0.11	Wang et al., 2013
13	Syn6803	Alk(a/e)nes	Ado-Aar		Air	ND	1.9	ND	Hu et al., 2013
14	Syn6803	Alk(a/e)nes	’TesA, ’FAP from *C. variabilis*	Aas	1% CO_2_, 300 μE/m^2^/s	111.2	77.1	0.46	Yunus et al., 2018
15	Syn7002	Alk(a/e)nes	Ado and Aar from Syn7942		5% CO_2_, 300 μE/m^2^/s	ND	7.5	ND	Knoot and Pakrasi, 2019
16	Ana7120	Alk(a/e)nes	Ado, Aar from *Aphanothece halophytica*		40 μE/m^2^/s, 140 mM NaCl	ND	1.3	ND	Kageyama et al., 2015
17	Npu73102	Alk(a/e)nes	Ado, Aar, Npun_F5141 (Lipase)		25°C, Air, 135∼160 μE/m^2^/s, MA medium	ND	129	ND	Peramuna et al., 2015
18	Syn6803	Fatty alcohols	Far from jojoba		5% CO_2_, 100 μE/m^2^/s	0.2	0.1^a^	0.00046	Tan et al., 2011
19	Syn6803	Fatty alcohols	Two copies of Far from jojoba, At3g11980 from *A. thaliana*		Air, 30∼50 μE/m^2^/s	ND	0.8	ND	Qi et al., 2013
20	Syn6803	Fatty alcohols	Maqu_2220 from *Marinobacter aquaeolei* VT8	Aar, Ado	Air, 50 μE/m^2^/s	1.3	2.9	0.0032	Yao et al., 2014
21	Syn6803	Fatty alcohols	Maqu_2220 from *M. aquaeolei* VT8	PlsX (Slr1510) transcriptionally inhibited by CRISPRi	1% CO_2_, 60 μE/m^2^/s	ND	10.4	ND	Kaczmarzyk et al., 2018
22	Syn6803	Fatty alcohols	’TesA, Sfp, CAR from *M. marinum*	Aas	1% CO_2_, 60 μE/m^2^/s	ND	68.0	ND	Yunus et al., 2018
23	Syn6803	Fatty alcohols	Tes3 from *A. tetradius*, Sfp, CAR from *M. marinum*	Aas	1% CO_2_, 60 μE/m^2^/s, overlaid with isopropyl myristate	100.0	80.0	0.42	Yunus and Jones, 2018
24	Syn6803	Fatty alcohols	Sfp, CAR from *M. marinum*	Aas	1% CO_2_, 60 μE/m^2^/s, overlaid with isopropyl myristate, with octanoic acid feeding	905.7^b^	ND	4.71	Yunus and Jones, 2018
25	Syn7942	FAEEs	AtfA from *A. baylyi* ADP1, Pdc and Adh from *Zymomonas mobilis*, XpkA from *A. nidulans*, Pta from *B. subtilis*		5% CO_2_, 100 μE/m^2^/s, 20% hexadecane overlay	15.11	50.0	0.06	Lee et al., 2017
26	Syn7942	Wax	AtfA, Aar, Slr1192 from Syn6803 or ACIAD3612 from *A. baylyi* ADP1		1% CO_2_, 50 μE/m^2^/s	ND	ND	ND	Kaiser et al., 2013

*^a^The number was converted using a calibration between DCW and optical density (1.0 OD_750_ equals approximately 200 mg DCW/L) (Yao et al., 2014); ^b^octanoic acid titer; ^c^yield relative to biomass; ^s^for oleochemicals secreted out of cells, otherwise, for the total oleochemicals; ND, not determined.*

### Disruption of Fatty Acid Re-activation

There is no complete fatty acid β-oxidation pathway for FFA degradation in cyanobacteria, based on bioinformatics analysis. However, acyl–acyl carrier protein synthetases (Aas) were proven to be able to re-activate FFAs to acyl-ACPs (Figure 2), and the latter can be incorporated into the membrane lipids through the above-mentioned acyltransferases in cyanobacteria (Kaczmarzyk and Fulda, 2010). The disruption of Aas led to the FFA accumulation and secretion in both Syn6803 and *Synechococcus elongatus* PCC7942 (hereafter Syn7942) (Kaczmarzyk and Fulda, 2010). This strategy has been adopted by most research efforts on cyanobacterial FFA production (Liu et al., 2011a; Ruffing and Jones, 2012; Ruffing, 2014) (Table 1).

### Heterologous Metabolic Pathway Engineering Toward FFAs and Chain Length Control

Hydrolysis of acyl-ACP to FFA by thioesterases can release the feedback inhibition of acyl-ACP to some enzymes of FAS-II and has in turn been confirmed to be an effective strategy to enhance FFA production in *E. coli* (Heath and Rock, 1996a, b; Lu et al., 2008). However, there is no gene encoding a thioesterase in cyanobacteria. Engineering efforts for improving FFA production in cyanobacteria began nearly a decade ago (Liu et al., 2011a). In this work, a truncated *E. coli* TE gene *’tesA* and three plant TE genes were heterologously expressed in the *aas* mutant of Syn6803 to achieve the production and secretion of FFAs (Liu et al., 2011a). Other thioesterases from *Arabidopsis thaliana* (FatB) (Hu et al., 2013), *Chlamydomonas reinhardtii* (Fat1) (Ruffing, 2013a), *Acinetobacter baylyi* (’AcTesA) (Afrin et al., 2018), and *Arachis hypogaea* L. (AhFatA, AhFatB) (Chen et al., 2017) were also functionally expressed in cyanobacteria for FFA production (Table 1), yielding long-chain (C16–C18) FFAs in most cases.

Medium-chain fatty acids (MCFAs, C4–C12) are valuable precursors to gasoline, but are not typical products of microbial fatty acid synthesis (Torella et al., 2013). Different from the above-mentioned thioesterases, thioesterases from *Cinnamomum camphorum* (CcFatB1), *Umbellularia californica* (UcFatB1), *Cuphea hookeriana* (ChFatB1), and *Anaerococcus tetradius* (Tes3) prefer medium chain length acyl-ACP substrates. When producing MCFAs, they were expressed in cyanobacteria to control the chain lengths of the FFA products (Murata and Wada, 1995; Work et al., 2015; Yunus and Jones, 2018). It is noteworthy that these short or medium chain length specific thioesterases should be expressed in the *aas* mutant to avoid the reactivation and the elongation of FFAs (Table 1). In addition, the replacement of the native FabH with a *Chaetoceros* ketoacyl-ACP synthase III in the lauric acid-secreting strain of Syn7002 increased MCFA synthesis up to five-fold (Gu et al., 2016).

### Translocation of FFA Out of the Cells

Besides the activity of acyl–acyl carrier protein synthetase, cyanobacterial Aas was also identified as a FFA importer (von Berlepsch et al., 2012) (Figure 2). And the inactivation of Aas resulted in the FFA secretion in some cyanobacterial strains, such as Syn6803, Syn7942 (Kaczmarzyk and Fulda, 2010), and Syn7002 (Ruffing, 2014), indicating that FFAs can be exported out of cyanobacterial cells by active or passive transport. It was proved that weakening cell walls by the deletion of the possible surface protein (Sll1951) and the peptidoglycan assembly protein (PBP2) as well as by ampicillin treatment led to the decrease of intracellular FFA amounts and the increase of overall FFA production in Syn6803 (Liu et al., 2011a).

**FIGURE 3:**
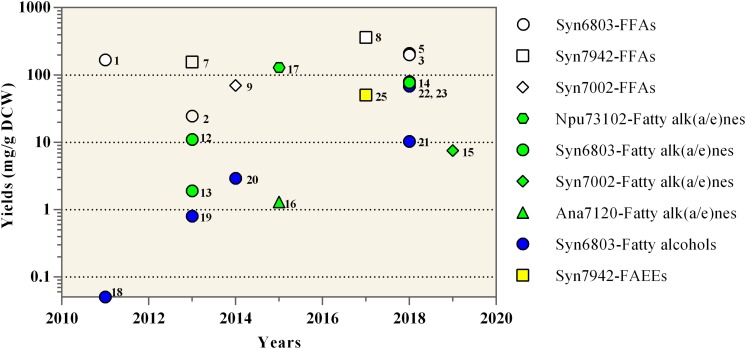
Yields of oleochemicals reported from engineered cyanobacteria. Maximum oleochemical yields in each study are shown at the years in which the study has been published. The detailed information for each data point including the reference is listed in Table 1 and can be retrieved using the adjacent number. The reported works on Syn6803, Syn7942, Syn7002, Npu73102, and Ana7120 are shown as circles, squares, diamonds, hexagons, and triangles, respectively. The work on the production of FFAs, Fatty alk(a/e)nes, Fatty alcohols, and FAEEs are shown as white, green, blue, and yellow colors, respectively.

Moreover, a RND-type FFA efflux system (RndA1B1) was identified by genomic analysis of a spontaneous mutant of the FFA-producing strain of Syn6803 (Kato et al., 2015) (Figure 2). Furthermore, the highest FFA yield (0.36 g/g dry cell weight) up to now has been achieved in the RndA1B over-expressing strain of Syn7942 through *in situ* removal of the FFA product from the culture medium by an isopropyl myristate (IM) overlay (Kato et al., 2017) (Figure 3). Recently, Sll0180 and Slr2131, homologs to AcrA and AcrB of *E. coli* respectively, were identified to be another FFA efflux system (Bellefleur et al., 2019) (Figure 2). Replacing the native *slr2131* with the *E. coli acrB* gene significantly increased the extracellular FFA concentration of Syn6803 (Bellefleur et al., 2019).

### Native Biosynthesis Pathway for Free Fatty Acids in Cyanobacteria

In fact, little FFAs was found in the cells of cyanobacteria grown under the normal culture condition (Kaczmarzyk and Fulda, 2010). Isotope labeling experiments indicated that they are released from membrane lipids (Kaczmarzyk and Fulda, 2010). Lipases were considered to be responsible for the releasing of FFAs from membrane lipids, and heterologous expression of the foreign lipase resulted in the increase of FFAs in Syn6803 (Liu et al., 2011b). *sll1969* is the only candidate lipase gene in the genome of Syn6803. The deletion of this gene decreased the FFA production, but did not completely block the FFA production (Gao et al., 2012a), suggesting it is not the only pathway for endogenous FFAs biosynthesis in Syn6803.

Besides the lipase, two cyanobacterial aldehyde dehydrogenases (AldE), namely Synpcc7942_0489 from Syn7942 (Kaiser et al., 2013) and Slr0091 from Syn6803 (Trautmann et al., 2013), were proven sufficient to oxidize fatty aldehyde precursors into fatty acids (Figure 2). It is noteworthy that these two aldehyde dehydrogenases are also able to utilize aldehyde substrates with shorter chain lengths (C8 to 12) (Kaiser et al., 2013) or apocarotenals (Trautmann et al., 2013), besides long-chain fatty aldehydes. For the purpose of FFA over-production in cyanobacteria, overexpression of acyl-ACP reductase (Aar) in the presence of AldE was proven to be a successful strategy (Kaiser et al., 2013) (Table 1).

### Over-Production of Polyunsaturated Fatty Acids by Introduction of Heterologous Desaturases

Syn7942 encodes only one Δ9 dedaturase gene (*desC*) in its genome and has only saturated and monounsaturated (Δ9) fatty acid chains in its membranes. Heterologous expression of the Δ12 desaturase gene (*desA*) from Syn6803 led to the conversion of endogenous monounsaturated fatty acids into dienoic fatty acids (Δ9, 12) and in turn changed the fatty acid compositions of Syn7942 (Wada et al., 1990). This modification further enhanced host tolerance to chilling (Wada et al., 1990) and strong light illumination (Gombos et al., 1997). In a recent study, the heterogeneous expression of two desaturases (DesA and DesB) from Syn7002 conferred an ability of producing alpha-linolenic acid (ALA; Δ9, 12, 15) to Syn7942 (Santos-Merino et al., 2018). Further, the ALA content of the desaturases-expressing mutant was improved to levels as high as 22.6% of the total lipids, by two metabolic engineering approaches designated as the *fabF* overexpression and the *fadD* disruption (Santos-Merino et al., 2018).

## Metabolism of Fatty Alk(a/e)nes in Cyanobacteria

Since 1960s, cyanobacteria were known to be able to naturally produce linear and branched fatty alkanes and alkenes with carbon chain lengths ranging from 15 to 19, besides membrane lipids (Han et al., 1968; Winters et al., 1969). However, the cyanobacterial biosynthesis pathways of fatty alk(a/e)nes were not identified until 2010 (Schirmer et al., 2010).

### Fatty Alk(a/e)nes Biosynthesis Pathways in Cyanobacteria

A two-enzyme pathway, consisting of acyl-ACP reductase (Aar) and aldehyde-deformylating oxygenase (Ado), was first identified by both comparative genomic and enzymatic analysis (Schirmer et al., 2010). In the Aar-Ado pathway, Aar catalyzes the conversion of acyl-ACP to fatty aldehydes, and Ado oxidizes and deformylates aldehydes to alk(a/e)nes (Figure 2), including pentadecane, heptadecane, 8-heptadecene, or 7-methylheptadecane.

A year later, the second native cyanobacterial alkene biosynthesis pathway (olefin synthase, Ols) was characterized in Syn7002 (Mendez-Perez et al., 2011). Harnessing a modular type I polyketide synthase (Ols), fatty acyl-ACP precursors are elongated and decarboxylated to synthesize terminal 1-alkenes (Figure 2), including 1-heptadecene, 1-non-adecene, or 1,14-nanadecadiene. It is worth noting that all alk(a/e)nes-producing cyanobacteria harbor only one of the two above-mentioned pathways but never both in nature (Coates et al., 2014; Klahn et al., 2014). However, it was proved that both Ols and Aar-Ado pathways can co-exist in one engineered marine cyanobacterium (Yoshino et al., 2015; Knoot and Pakrasi, 2019). And the Aar-Ado pathway, as well as two non-cyanobacterial alkane biosynthesis genes, can complement an Ols knockout strain of Syn7002 (Knoot and Pakrasi, 2019).

### Physiological Effects of Alk(a/e)nes in Cyanobacteria

Despite the fact that alkane biosynthesis pathways were characterized, little is known about the physiological roles of alk(a/e)nes in cyanobacteria. Alkanes were shown to accumulate in thylakoid and cytoplasmic membranes of Syn6803 (Lea-Smith et al., 2016) as well as in lipid droplets of Npu73102 (Peramuna and Summers, 2014; Peramuna et al., 2015). Through reverse genetic approaches, it was found that cyanobacterial alkanes might play roles in regulating redox balance and reductant partitioning in photosynthesis (Berla et al., 2015), and modulating membrane flexibility, which is required for optimal cell division, size, and growth (Lea-Smith et al., 2016). In addition, alkanes were proven to be required for cyanobacterial tolerance to abiotic stresses including cold (Berla et al., 2015) and salt (Yamamori et al., 2018).

## Engineering Cyanobacteria to Produce Fatty Alk(a/e)nes

With the carbon chain lengths ranging from C15 to 19, cyanobacterial alk(a/e)nes could be directly used in diesel and jet engines, and have attracted great attention from academics. On the one hand, chemical structures and profiles of cyanobacterial alk(a/e)nes were examined across a wide range of cyanobacterial species (Liu et al., 2013; Coates et al., 2014; Zhu et al., 2018). On the other hand, several model cyanobacterial species were metabolically engineered for improving their alk(a/e)nes production (Mendez-Perez et al., 2011; Hu et al., 2013; Wang et al., 2013; Kageyama et al., 2015; Peramuna et al., 2015) (Table 1). Among these engineering approaches, the over-expression of endogenous or heterogeneous alk(a/e)ne biosynthesis genes was widely used and proven to be successful (Xie et al., 2017). In addition, the cyanobacterial alk(a/e)ne production can be further improved by increasing the copy numbers of these genes through inserting them into different genomic loci (Wang et al., 2013).

As expected, the over-expression of the multi-subunit acetyl-CoA carboxylase, which catalyzes the first step of fatty acid biosynthesis, was proven to be an effective way to enhance the cyanobacterial alkane production (Tan et al., 2011; Wang et al., 2013). The cyanobacterial alkane production can also be improved by blocking the competing pathway, like the poly-β-hydroxybutyrate (PHB) pathway (Wang et al., 2013). Different from cyanobacterial FFA production, Aas is beneficial for cyanobacterial alkane production in the Aar-Ado pathway (Gao et al., 2012b). The over-expression of Aas promoted cyanobacterial alkane production, because the acyl-ACP precursors of the Aar-Ado pathway are mainly from the Aas-mediated reactivation of FFAs, which are from the hydrolysis of membrane lipids rather than the *de novo* fatty acid biosynthesis pathway (Gao et al., 2012b). Similarly, the over-expression of lipolytic enzymes, which release the FFA by hydrolyzing membrane lipids, was also beneficial for alkane production (Wang et al., 2013; Peramuna et al., 2015). For example, the heptadecane production in Npu73102 was significantly improved by over-expression of Aar, Ado, and a lipase candidate Npun_F5141, together with the high light illumination, reaching 12.9% of dry cell weight (DCW) (Peramuna et al., 2015) (Figure 3).

In addition to the above engineering approaches on cyanobacterial native alkane biosynthesis pathways, several synthetic metabolic pathways were recently constructed and evaluated for alkane production in cyanobacteria (Yunus et al., 2018; Knoot and Pakrasi, 2019). In brief, three newly identified fatty acid decarboxylases were heterologously expressed in cyanobacteria successfully for converting FFA precursors to C_*n–*__1_ alk(a/e)ne end-products (Figure 2), including UndA (Rui et al., 2014) and UndB (Rui et al., 2015) from *Pseudomonas fluorescens* Pf-5, together with fatty acid photodecarboxylase (FAP) from *Chlorella variabilis* (Sorigue et al., 2017). Although catalyzing similar reactions with another two 1-alkenes producing enzymes, namely Ols from *Synechococcus* (Mendez-Perez et al., 2011) and OleTJE from *Jeotgalicoccus* (Rude et al., 2011), both UndA and UndB prefer FFA substrates with medium-chain lengths (C10–C16) rather than long-chain substrates (Rui et al., 2014, 2015). Recently, a phenylalanine 239 to alanine mutation of UndA (UndA-F239A) increased its enzymatic activities toward long chain fatty acids and improved its compatibility with cyanobacterial fatty acid compositions (Knoot and Pakrasi, 2019). FAP from eukaryotic algae mediates the light-driven conversion of fatty acid substrates to alkanes, with a wide range of substrate chain lengths (C12 to C18) and a higher substrate specificity to hexadecanoic acid (Sorigue et al., 2017). When expressing in the *aas* mutant of Syn6803 harboring a truncated *E. coli* thioesterase ’TesA, FAP can markedly improve alkane production (Yunus et al., 2018). And the total alkane yield was further increased to 77.1 mg/g DCW through the removal of the chloroplast transit peptide of FAP (’FAP) and the increase of light illumination (Yunus et al., 2018) (Table 1 and Figure 3).

## Engineering Cyanobacteria to Produce Fatty Alcohols

Fatty alcohols can be used in the manufacture of cosmetics, detergents, lubricants, and potentially as biofuels (d’Espaux et al., 2017). Similar to several reports on microbial production of fatty alcohols (Steen et al., 2010; d’Espaux et al., 2017), cyanobacterial fatty alcohol production (Table 1) was mainly realized by heterologous expression of fatty acid reductases (Far) which utilize fatty acyl-ACP or acyl-CoA as substrates and NADH or NADPH as cofactors (Tan et al., 2011; Yao et al., 2014; Kaczmarzyk et al., 2018). All the Fars that worked well in cyanobacteria are from plants (Tan et al., 2011) and bacterium (Yao et al., 2014), whereas cyanobacteria expressing the Fars from mice failed to produce any fatty alcohols (Tan et al., 2011). The engineered strain of Syn6803 harboring the Far from jojoba (*Simmondsia chinensis*) produced only 0.05 mg fatty alcohols per gram DCW (Tan et al., 2011). Then, the cyanobacterial fatty alcohol yield was dramatically improved to 0.76 mg/g DCW by increasing the copy numbers of the plant Far-expressing cassettes and blocking both the PHB and glycogen biosynthesis pathways (Qi et al., 2013) (Table 1).

Compared to the plant Fars, a bacterial Far (Maqu_2220 from *Marinobacter aquaeolei* VT8) showed better substrate preferences to long-chain fatty acyl CoA/ACP (C16–C18) (Hofvander et al., 2011) and better performance in the engineered strain of Syn6803 for fatty alcohol production (Yao et al., 2014). For further improving fatty alcohol production of this Maqu_2220-expressing strain, the inactivation of the fatty alkane biosynthesis pathway which competes with Far for acyl-ACP precursors was shown to be effective, resulting in 2.9 mg/g DCW fatty alcohols (Yao et al., 2014). Recently, phosphate acyltransferase PlsX was identified as another key node in C18 fatty acyl-ACP consumption, and the fatty alcohol yield of Syn6803 was increased to 10.4 mg/g DCW by transcriptional inhibition of *plsX* using CRISPR-interference (CRISPRi) technique (Kaczmarzyk et al., 2018) (Table 1).

Besides Fars, carboxylic acid reductase (CAR) from *Mycobacterium marinum*, which can effectively convert FFAs into corresponding fatty aldehydes, was also used for cyanobacterial fatty alcohol production (Yunus and Jones, 2018; Yunus et al., 2018). In the presence of Ado, heterologous expression of CAR in the FFA-producing strain of *Syn*6803 unexpectedly led to the conversion of most of the FFA pool into corresponding fatty alcohols rather than fatty alk(a/e)nes, resulting in ∼68 mg/g DCW fatty alcohols (Yunus et al., 2018). It was speculated that the Ado failed in competition with native aldehyde reductases (or alcohol dehydrogenases, Adh) (Yunus et al., 2018). For producing medium chain-length fatty alcohols, Tes3 from *A. tetradius*, rather than *E. coli* ’TesA was co-expressed in the *aas* mutant of Syn6803 together with CAR and its maturation protein Sfp. Through the optimization of promoters and ribosomal binding sites and *in situ* product extraction with isopropyl myristate, the titers of 1-octanol and 1-decanol of the above mutant were increased to more than 100 mg/L, which is the highest titer of cyanobacterial fatty alcohols to date (Yunus and Jones, 2018) (Table 1). However, it should be noted that this 1-octanol producing strain displayed genetic instability and reduced 1-octanol production during continuous sub-culturing (Yunus and Jones, 2018).

## Engineering Cyanobacteria to Produce Fatty Acid Esters

Industrially, fatty acid esters are produced by transesterification of vegetable oils or animal fats with an alcohol in the presence of a suitable catalyst. For microbial production of fatty acid esters, the transesterification process is performed enzymatically by a multi-functional wax ester synthase/acyl-CoA:diacylglycerol acetyl transferase (WS/DGAT) (Janssen and Steinbuchel, 2014). Due to its wide substrate specificities to alcohols with various carbon lengths, the WS/DGAT (AtfA) from *Acinetobacter baylyi* ADP1 was normally used in cyanobacteria to mediate the combination of the activated fatty acids with ethanol (Lee et al., 2017) or fatty alcohols (Kaiser et al., 2013) (Table 1). To achieve wax ester production in Syn7942, AtfA was co-expressed with the Aar as well as a long-chain alcohol dehydrogenase from Syn6803 (Adh, Slr1192) or *A. bayli* (ACIAD3612) (Kaiser et al., 2013). Given the fact that FFA accumulates in the wax-producing strain as the byproduct (Kaiser et al., 2013), the endogenous *aldE* gene might be a candidate target for improving wax production by metabolic engineering.

For fatty acid ethyl esters (FAEEs) production in Syn7942, an ethanol biosynthesis pathway was firstly constructed by introducing both pyruvate decarboxylase and alcohol dehydrogenase from *Zymomonas mobilis* (Lee et al., 2017). The further expression of the AtfA in the ethanol-producing strain resulted in 40% ethanol reduction and the detection of trace concentrations of palmitic acid ethyl ester (Lee et al., 2017). A synthetic phosphoketolase pathway containing a phosphoketolase from *A. nidulans* (XpkA) and a phosphotransacetylase from *B. subtilis* (Pta) was then introduced to increase the acetyl-CoA pool and the FAEE production (Lee et al., 2017). The FAEE production was finally increased further to 50.0 mg/g DCW by culture optimization (Lee et al., 2017) (Figure 3). However, the low substrate specificities of the WS/DGAT to ethanol (Stoveken et al., 2005) could be a bottleneck for the FAEE production, considering the appearance of ethanol byproducts.

## Redirecting Carbon Flux to Oleochemical Biosynthesis Pathways

Increasing the substrate supply and blocking the competitive pathways are routine strategies for improving the production of target products. Different from heterotrophic microbes, autotrophic cyanobacteria utilize CO_2_ rather than sugars as carbon sources, using the Calvin-Bassham-Benson (CBB) pathway. Thus, the RuBisCO, which is the key enzyme in the CBB cycle, has been considered as an ideal target for improving cyanobacterial carbon fixation. Over-expression of the RuBisCO from Syn7942 in Syn7002 led to a more than three-fold increase in FFA production (Ruffing, 2014). However, the same strategy did not work to improve the FFA production of Syn7942 (Ruffing, 2013a).

For directing the fixed carbon flux to *de novo* fatty acid biosynthesis pathway, a heterologous phosphoketolase pathway, which was discovered to be efficient for increasing the supply of acetyl-CoA precursor, was introduced into the FAEE-producing strain of Syn7942, and greatly enhanced the FAEE production (Lee et al., 2017). In addition, over-expression of the ACCase has been considered to direct the carbon flux into the fatty acid pathway, and was confirmed effective for FFA over-production in *E. coli* (Lu et al., 2008). However, it did not always work for improving FFA production of cyanobacteria. For example, the over-expression of an ACCase from *C. reinhardtii* led to a 20% increase of the specific FFA production in the FFA-producing strain of Syn7942 (Ruffing, 2013a) and a 56% increase of total fatty alk(a/e)nes in Syn6803 (Tan et al., 2011) (Table 1), but there was no significant change of FFA yield in Syn6803 using a similar approach (Liu et al., 2011a).

As a major carbon sink, glycogen can account for more than 50% of the DCW in some cyanobacteria treated by the stressed conditions (Song et al., 2016), which indicates that glycogen biosynthesis competes with the fatty acid biosynthesis pathway for carbon flux. However, the complete disruption of the glycogen biosynthesis always resulted in an increase of cyanobacterial susceptibility to stress conditions (Luan et al., 2019), and the reconfiguration of electron flow in photosynthesis (Work et al., 2015). Thus, the deletion of *glgC*, the key gene for cyanobacterial glycogen biosynthesis, showed a slight increase of fatty alcohol production in Syn6803 (Qi et al., 2013) and no increase of lauric acid production in Syn7002 (Work et al., 2015).

## Improving Cyanobacterial Tolerance to Oleochemicals

As hydrophobic compounds, oleochemicals inevitably interact with cell membranes, which are sites of photosynthesis and respiration, and will result in a series of physiological effects, including reduced photosynthetic yields, chlorophyll-a degradation, changes in the cellular localization of the light-harvesting pigments (Ruffing and Jones, 2012), increased reactive oxygen species (ROS), cell membrane permeability (Ruffing, 2013b), and impaired cell growth (Kamarainen et al., 2012; Ruffing and Jones, 2012).

A dozen candidate genes were identified by comparative transcriptome analyses with potentials to mitigate FFA toxicity. The disruption of two porins and the overexpression of ROS-degrading proteins were confirmed to be effective in reducing the toxic effects of FFA production and recovering cell growth (Ruffing, 2013b). Furthermore, transporters specific to oleochemicals are promising candidates to secrete oleochemicals out of cells. The inactivation of Aas, which also functions as a FFA uptake transporter, was found to be able to alleviate FFA toxicity of cyanobacteria (von Berlepsch et al., 2012). Recently, it was found that the over-expression of a RND-type FFA exporter (RndA1B1) or the native or foreign (AcrAB) efflux systems for FFAs enhanced FFA secretion and cell growth (Kato et al., 2015; Bellefleur et al., 2019). Besides transporters, *in situ* removal of oleochemicals from the culture medium by some organic solutes was demonstrated to be able to significantly increase cyanobacterial cell tolerance to fatty alcohol, FFA, or FAEE (Kato et al., 2017; Lee et al., 2017; Yunus and Jones, 2018).

## Discussion

In the past decade, cyanobacteria were successively engineered to produce oleochemicals directly from CO_2_, inspired by successful strategies on oleochemical production by *E. coli*. Although these efforts proved the concept of engineering cyanobacteria for oleochemical production, the engineered strains are far from being used for commercial applications mainly due to their poor production ability. To improve cyanobacterial oleochemical production, more intensive efforts are needed in the future.

As shown in Figure 2, all fatty acyl chains of oleochemicals are ultimately from the *de novo* fatty acid biosynthesis pathway in cyanobacteria. The fundamental works on the regulatory mechanisms of cyanobacterial fatty acid metabolism are required to identify potential targets for unlocking and boosting fatty acid biosynthesis. Excretion of oleochemicals out of cells by transporters can alleviate the toxicity of FFA and was shown to be an effective strategy for improving cyanobacterial FFA production (Kato et al., 2015, 2017). However, native or foreign transporters for other oleochemicals still need to be discovered and evaluated in cyanobacteria. In addition, it should be helpful for improving cyanobacterial production of oleochemicals by introducing a heterologous reductant regenerating system to balance the reductant generation and utilization, considering the fact that reductant is needed in in oleochemical biosynthesis pathways.

Moreover, genetic instability of engineered cyanobacteria (Jones, 2014) is another potential issue for future commercial application. Despite limited observation up to now (Takahama et al., 2003; Jones, 2014), genetic instability can randomly result in some mutations on the genes associated with the production traits. And these mutations will be enriched and finally lead to the failure of cyanobacterial production of some toxic compounds like oleochemicals, if they are beneficial for fitness. For reliable production of oleochemicals in engineered cyanobacteria, key genes associated with genetic fidelity should be identified though systematic genetic analysis and then modified in cyanobacteria to construct the chassis with a higher genetic stability. On the other hand, some inducible promoters or novel genome editing tools should be used to drive the oleochemical biosynthesis only when cyanobacterial cells stop to propagate, reducing the chance of spreading the mutation through the population in long-term culturing.

## Author Contributions

XT and SY conceived the outline and revised the manuscript. LW, LC, and XT drafted the manuscript. All authors read and approved the final manuscript.

## Conflict of Interest

The authors declare that the research was conducted in the absence of any commercial or financial relationships that could be construed as a potential conflict of interest.

## Abbreviations

***Enzymes:*** Aar: acyl-ACP reductase
Aas: acyl–acyl carrier protein synthetases
ACCase: acetyl-CoA carboxylase
ACP: acyl carrier protein
Adh: alcohol dehydrogenase
Ado: aldehyde deformylating oxygenase
AldE: aldehyde dehydrogenase
AtfA: wax ester synthase/acyl-CoA:diacylglycerol acetyl transferase from *Acinetobacter baylyi* ADP1
CAR: carboxylic acid reductase
FabD: malonyl-CoA:ACP transacylase
FabF: *b*-ketoacyl -ACP synthase II
FabG: β-ketoacyl -ACP reductase
FabH: β-ketoacyl -ACP synthase III
FabI: enoyl-ACP reductase
FabZ: β-hydroxyacyl -ACP dehydrase
FAP: fatty acid photodecarboxylase (FAP)
Far: fatty acid reductase
LipA: lipase
Ols: olefin synthase
Pdc: pyruvate decarboxylase
PlsC: lysophosphatidic acid acyltransferase
PlsX: phosphate acyltransferase
PlsY: acylglycerol-phosphate acyltransferase
RndA1B1: a cyanobacterial RND-type efflux system
RuBisCO, ribulose 1: 5-bisphosphate carboxylase/oxygenase
Sfp: phosphopantetheinyl transferase
TE: thioesterase
UndA, UndB: fatty acid decarboxylases from *Pseudomonas* sp.
***Compounds:*** FAEEs: fatty acid ethyl esters
FFAs: free fatty acids
IM: isopropyl myristate
LPA: lysophosphatidic acid
PA: phosphatidic acid
PHB: poly-β-hydroxybutyrate
PYR: pyruvate.
